# Altered functional connectivity in children born very preterm at school age

**DOI:** 10.1038/s41598-022-11184-x

**Published:** 2022-05-04

**Authors:** Hye Jung Cho, Hyejin Jeong, Chan-A Park, Dong Woo Son, So-Yeon Shim

**Affiliations:** 1 Division of Neonatology, Department of Pediatrics, Gil Medical Center, Gachon University College of Medicine, Incheon, Korea; 2 Neuroscience Convergence Center, Green Manufacturing Research Center, Korea University, Seoul, Korea; 3 Biomedical Engineering Research Center, Gachon University, Incheon, Korea; 4 Division of Neonatology, Department of Pediatrics, School of Medicine, Ewha Womans University, Seoul, Korea

**Keywords:** Intelligence, Neuronal development

## Abstract

Children born very preterm are at significant risk of neurodevelopmental impairment. This study sought to identify differences in cognitive function in children born very preterm compared to term-born controls and investigate alteration in white matter microstructure and functional connectivity (FC) based on tract-based spatial statistics (TBSS) and resting-state functional MRI, respectively. At 6 years of age, 36 children born very preterm (< 32 weeks' gestation) without major neurological disabilities and 26 term-born controls were tested using the Wechsler Intelligence Scale for Children, 4th edition, and Child Behavior Checklist. Whole-brain deterministic tractography and FC measurements were performed in both groups. The very preterm group had significantly lower intelligence scores than the term-born controls. The TBSS revealed no significant differences between the two groups, whereas FC was significantly increased between the frontoparietal network and the language network and was significantly decreased between the right salience network nodes in the very preterm group. The altered FC patterns between specific regions of the higher-order networks may reflect underlying deficits in the functional network architecture associated with cognitive function. Further studies are needed to demonstrate a direct connection between FC in these regions and cognitive function.

## Introduction

Very preterm birth is defined as a birth before the completion of 32 weeks'gestation^1^. Advances in prenatal care have led to an increase in the survival rates of very preterm infants, and the incidence of major disabilities including cerebral palsy, mental retardation, deafness, or blindness have been improved^2,3^. However, surviving children born very preterm are at risk of developing problems with cognitive function, including intelligence, learning and memory, lower academic performance, and behavioral problems, which persist throughout childhood and young adulthood^4–6^. Early school age, 6 years of age, is a crucial period because children start reading and various academic programs at this age.

Early investigations have employed conventional magnetic resonance imaging (MRI) and diffusion tensor imaging (DTI) to characterize alterations in cerebral structural development associated with preterm birth^7,8^. Preterm infants have reduced white matter (WM) and grey matter volume and altered diffusion properties from infancy compared to term-born infants^9,10^. Regarding cognitive outcomes, children born very preterm were found to have associations between reduced microstructural connectivity within widespread networks with impaired cognitive abilities^11,12^. However, these studies do not completely explain the cognitive difficulties of preterm children without overt brain lesions^13^. For this population, resting-state functional MRI (rs-fMRI) may provide clues regarding abnormal development.

rs-fMRI has been less well-studied than other imaging modalities in the preterm population. rs-fMRI is a powerful, non-invasive tool with high sensitivity for delineating alterations in the developing brain^14,15^. Specifically, rs-fMRI is used to detect temporal correlations in spontaneous, low-frequency fluctuations in blood oxygen level-dependent (BOLD) signal, which occurs between functionally-related brain regions independent of task^16^. Resting-state functional connectivity (FC) is one of the most commonly used indicators to reflect functional connections between brain regions. Prior studies on the association between resting-state FC and intelligence showed that the interaction between association cortices within the parietal and frontal brain regions is important for differences in intelligence^17,18^. Furthermore, previous studies on preterm populations have suggested that preterm birth leads to changes in the resting-state network (RSN) development^13,19^. However, the long-term effects of preterm birth on RSN architecture and their role in impaired neurodevelopmental outcomes remain not yet fully established^13^. Further understanding of the neurodevelopmental sequelae of preterm birth requires more advanced investigations of the underlying neurobiological mechanisms that support brain development.

To our knowledge, there has been no study that analyzed microstructural alterations and FC simultaneously in children born very preterm and term-born controls at 6 years of age. This study sought to compare cognitive and behavioral development at 6 years between children born very preterm and term-born controls using the Wechsler Intelligence Scale for Children, 4th edition (WISC-IV), and the Child Behavior Checklist (CBCL). Using DTI and rs-fMRI in the same study population, we aimed to investigate the effect of prematurity on microstructural alterations and region of interest (ROI)-to-ROI FC across the whole brain. The present study may increase our understanding of the long-term impact of very preterm birth on functional network architecture and cognitive function.

## Results

Of the 67 children (40 very preterm and 27 term-born), two very preterm children failed MR scanning. For two very preterm and one term-born subjects, data were discarded because of excessive motion artifacts. Finally, 36 very preterm and 26 term-born children successfully underwent MRI scans and neurodevelopmental assessments. No other brain abnormalities were seen on T1-MRI images. The mean gestational age in the very preterm group was 27.5 ± 2.4 weeks, and the mean birth weight was 1086.9 ± 350.5 g. All term-born participants were born after 37 weeks' gestation and weighed > 2500 g at birth. There were no significant differences in age at study or sex distribution between the very preterm group (mean age: 76.1 ± 3.9 months, 22 males) and the term-born group (mean age 80.1 ± 3.1 months, 14 males). The demographic and clinical characteristics are presented in Table 1.

**Table 1:** Demographic and clinical characteristics.

Characteristic	Very preterm (*n* = 36)	Term (*n* = 26)	*P*
Gestational age (wk)	27.5 ± 2.4	39.5 ± 0.3	< 0.001
Birth weight (g)	1086.9 ± 350.5	3357.0 ± 371.4	< 0.001
Male	22 (61.1)	14 (53.8)	0.570
Age at study (mo)	76.1 ± 3.9	80.1 ± 3.1	0.180
Intraventricular hemorrhage (grade 1)	18 (50)		
Respiratory distress syndrome	20 (55.6)		
Patent ductus arteriosus requiring surgery or medication	22 (61.1)		
Bronchopulmonary dysplasia (≥ moderate)	12 (33.3)		
Necrotizing enterocolitis (≥ stage 2)	7 (61.1)		
Proven sepsis	3 (8.3)		

Data are presented as mean ± standard deviation for continuous variables and *n* (%) values for categorical variables.

### Neurodevelopmental outcomes

The results of the neurodevelopmental assessment are presented in Table 2. Children born preterm had significantly lower scores on all included subscales of the WISC-IV than term-born controls. After adjusting for multiple comparisons, there were no significant differences in the CBCL scores between the children born very preterm and controls.

**Table 2:** Comparison of neurodevelopmental outcomes.

Test	Very preterm (*n* = 36)	Term (*n* = 26)	*P*
**WISC-IV**			
Verbal comprehension index	89.64 ± 15.94	99.83 ± 12.90	0.013
Perceptual reasoning index	87.47 ± 16.36	101.70 ± 12.54	0.001
Working memory index	86.53 ± 17.19	97.13 ± 11.00	0.005
Processing speed index	86.44 ± 18.34	98.30 ± 15.29	0.013
FSIQ	83.94 ± 15.89	98.65 ± 10.85	< 0.001
**CBCL**			
Anxious/depressed	56.69 ± 5.33	56.23 ± 8.12	0.851
Withdrawn/depressed	56.33 ± 6.35	54.62 ± 4.90	0.327
Somatic complaints	56.03 ± 6.58	56.31 ± 7.41	0.906
Social problems	61.31 ± 9.88	55.85 ± 7.79	0.055
Thought problems	58.44 ± 6.86	56.85 ± 6.44	0.459
Attention problems	57.61 ± 8.35	53.08 ± 4.70	0.023
Rule-breaking	57.83 ± 6.54	54.77 ± 3.96	0.055
Aggressive behavior	58.22 ± 8.92	53.62 ± 5.85	0.044
Internalizing problems	56.50 ± 6.69	53.46 ± 10.96	0.332
Externalizing problems	57.92 ± 11.09	51.08 ± 7.75	0.026
Total problems	58.39 ± 8.87	52.08 ± 10.01	0.059

Data are shown as mean ± standard deviation.

*CBCL* Child behavior checklist, *FSIQ* full-scale intelligence quotients, *WISC-IV* Wechsler Intelligence Scale for Children, Fourth Edition.

### DTI and ROI-to-ROI FC between the two groups

**Figure 1:**
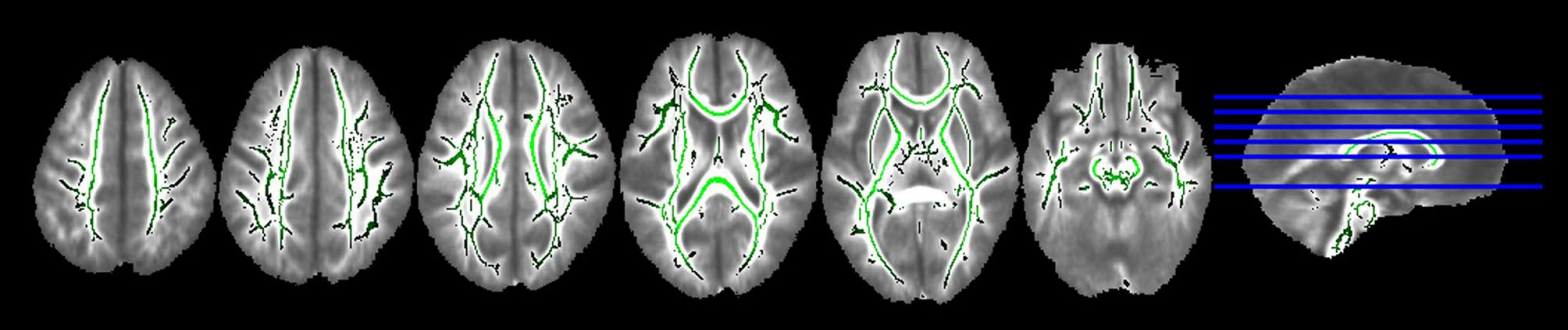
Comparisons of the mean FA maps between two groups. The mean FA skeleton is shown in green, and there were no significant differences between the two groups. FA, fractional anisotropy.

**Figure 2:**
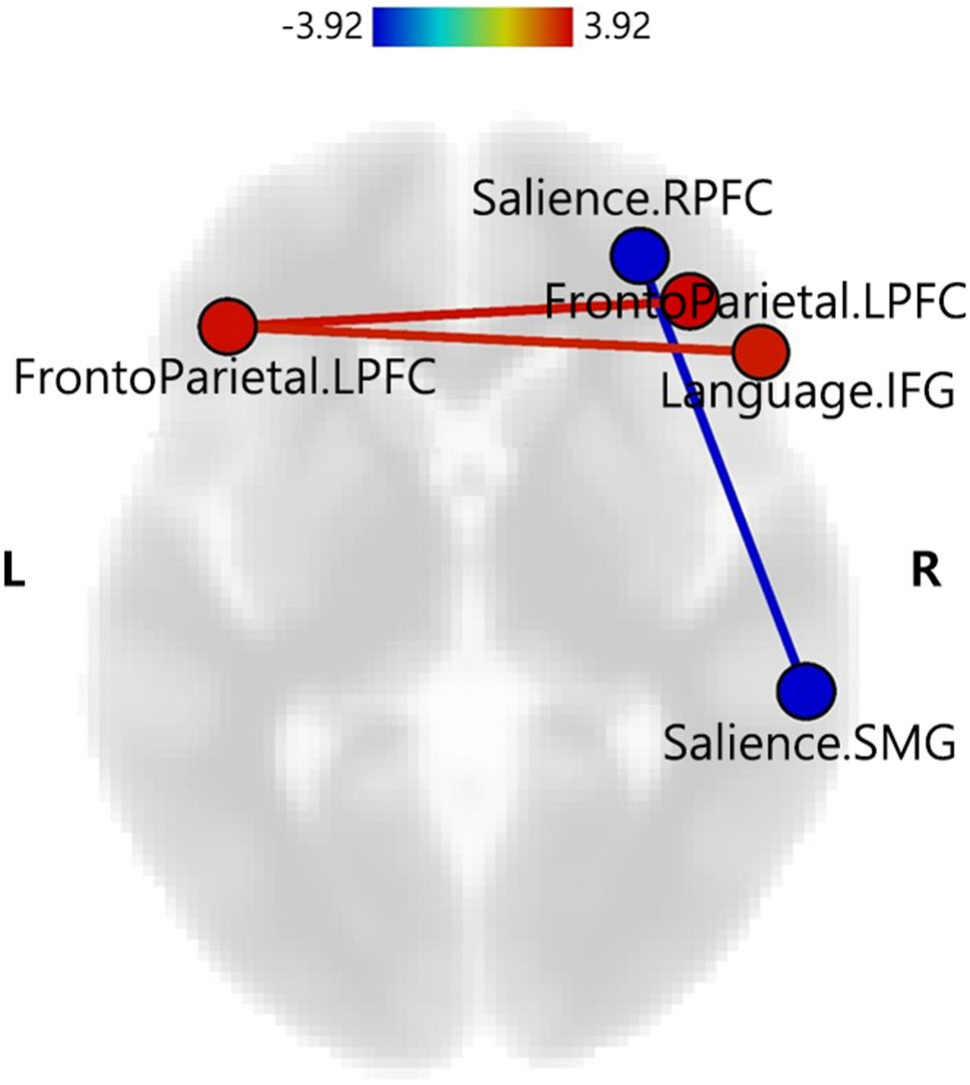
Significant group differences in ROI-to-ROI FC between two groups. Red lines indicate connections with increased functional connectivity in the preterm group, and the blue line indicates the connection with decreased functional connectivity in the preterm group compared to the term group. The results are presented at a threshold FDR of *P* < 0.05, with two-sided seed level correction. The color bar indicates the statistical *t-*value. FC, functional connectivity; IFG, inferior frontal gyrus; L, left; LPFC, lateral pre-frontal cortex; R, right; ROI, region of interest; RPFC, rostral pre-frontal cortex; SMG, supramarginal gyrus.

The tract-based spatial statistics (TBSS) results are shown in Fig. 1. There was no significant difference in the development of WM microstructure between the two groups. Figure 2 illustrates the differences in ROI-to-ROI FC between the two groups. The very preterm group showed increased FC between the left lateral pre-frontal cortex (LPFC) of the frontoparietal network (FP) and the right LPFC of the FP and the right inferior frontal gyrus (IFG) of the language network (LAN) and decreased FC between the right rostral pre-frontal cortex (RPFC) and right supramarginal gyrus (SMG) of the salience network (SN). The corresponding *t*-values are listed in Table 3.

**Table 3:** ROI-to-ROI results showing functional connectivity differences between very preterm and term groups.

Pair connection (very preterm group > term group)	*t*	*P*
Lateral prefrontal cortex L–Lateral prefrontal cortex R	3.28	0.0452
Lateral prefrontal cortex L–Inferior frontal gyrus R	3.11	0.0452
Rostral prefrontal cortex R–Supramarginal gyrus R	− 3.45	0.0328

Threshold ROI-to-ROI connections by intensity, false discovery rate of *P* < 0.05, two-sided. L, left; R, right.

## Discussion

To the best of our knowledge, this is the first study to investigate microstructural connectivity based on DTI and FC on rs-fMRI at the whole-brain level in children born very preterm and term-born control at school age. Previous studies have reported that minor neurodevelopmental impairments may not become apparent until school age^20,21^, and this study supports this concept. Intelligence is an important and frequently used measure of cognitive function and is related to executive function, which are essential factors involved in academic difficulties and behavioral problems^22,23^. A previous meta-analysis showed that prematurity has an adverse effect on intelligence and attention problems in children born very, moderately, and late preterm, which persists until after school age^24^.

In this study, intelligence scores, including verbal comprehension, perceptual reasoning, working memory, and processing speed, were lower in very preterm children than in term-born controls. These findings are consistent with those of previous studies^24,25^. Several studies have also reported a relationship between WM microstructural alteration and adverse cognitive outcomes in preterm children^26,27^. However, despite significant differences in intelligence scores, we did not find any significant differences in the TBSS between children born very preterm and term-born controls. Previously, we demonstrated delayed entire WM microstructure in preterm neonates compared with term-born controls; however, at 1 year of age, WM development, except for that of the corpus callosum, had reached the development level of the term-born controls^28,29^. The present study is an extension of our previous studies, and we suggest that the growth of WM microstructure in preterm infants can catch up with that in term-born controls by school age. Some studies have shown that microstructural connectivity may be improved through education and intervention^30,31^. Children born preterm often experience selective cognitive impairment, even in the absence of overt brain lesions^32,33^. However, DTI may provide only limited structural information to elucidate changes in cerebral development that may lead to borderline cognitive deficits and minor behavioral problems at high risk of poor school performance. Microstructural connectivity and FC within RSNs are not necessarily identical^34,35^. This has led to the increasing utilization of state-of-the-art rs-fMRI, which provides information about brain maturity and integrity, and changes in resting-state FC with age^36^.

Primary networks located in cortical regions, such as the sensorimotor network (SMN) and visual network (VIS), are known to mature early and are less affected by premature birth^37,38^. In contrast, the higher-order networks, such as the FP and SN, are located in higher-order association cortices involved in controlling emotion regulation, attention, and cognition and are quantifiably vulnerable, demonstrating more significant inter-subject variability^39,40^. The results from the whole-brain ROI-to-ROI functional analysis showed that the very preterm group had altered resting-state FC within or between the regions of higher-order networks (FP, LAN, and SN), as compared with term-born controls, whereas no significant differences were found in primary networks.

The present study found that the FC of the left LPFC in the FP was increased reciprocally with that of the right LPFC and the IFG of the LAN in children born very preterm compared to term-born controls. The FP is the central executive network^41,42^. The pre-frontal cortex of the FP is associated with higher-level cognitive processes, including the organization of input from sensory modalities, maintenance of attention, monitoring of information in working memory, and coordination of goal-directed behaviors^43^. Recently, several rs-fMRI studies in children and adolescents born preterm have shown increased FC within and between the LAN, as well as between the LAN and other regions throughout the brain, resulting in relatively poor language development compared to term-born controls^44,45^. Those results might explain why the very preterm group showed lower verbal comprehension index scores than term-born controls in the present study. In contrast, the very preterm group showed decreased FC between the LPFC and SMG in the right SN regions. The SN is thought to facilitate the detection of relevant internal or environmental stimuli and assist appropriate behavioral responses^46^. The SN is known to be associated with broad psychopathological dimensions, particularly externalizing behaviors^47^. Our findings suggested that the long-lasting aberrant connectivity of the SN may be linked to a higher incidence of behavioral problems in preterm children, although there was no significant difference in behavioral scores in this study. Consistent with the present study, a recent review showed that older children born preterm could possess both increased and decreased FC compared with term controls^48^. Several studies have reported that preterm neonates show a systematic decrease in FC, which persists into early childhood, adolescence, and early adulthood^42,45,49^. The decreased FC found in children born very preterm could be interpreted as the result of long-lasting detrimental effects of preterm birth on intrinsic brain network connectivity^42^. However, other recent investigations have found increased FC patterns in preterm children that potentially correspond to poorer neurological outcomes^44,50,51^. Given the developmental trajectories suggested by prior studies, the increased FC in preterm children can most likely be explained by a compensatory effect against a decreased number of crossing fibers or thinner axons caused by the disruption of normal synaptic pruning that is expected to occur during childhood^44,51^.

The present study has several strengths. First, we included school-aged children, which is vital to addressing questions regarding long-term outcomes and academic achievement. Specifically, we combined modalities to elucidate the pathophysiology of brain development in preterm children by demonstrating microstructural connectivity and FC based on DTI and rs-fMRI, respectively. We included only children with normal brain MRI findings to avoid confounding effects of brain lesions on FC.

This study also has several limitations. First, the sample size was relatively small, with only 62 children. We did not assess the correlation between resting-state FC and cognitive function and could not provide information on the direct relationships between specific resting-state FC properties and cognitive function. Lastly, we were not able to investigate the effects of clinical variables, such as sex, gestational age, genetic or environmental factors, and postnatal exposure to stressors during the neonatal intensive care unit (NICU) hospitalization. However, the effects of such clinical variables on cognitive function have been reported in a previous well-designed study^22^.

In conclusion, we found that children born very preterm without apparent brain injury at school age had lower intelligence scores and exhibited persistent alterations in FC between specific regions of the RSNs located in higher-order association cortices compared with term-born controls. This study suggests that rs-fMRI can be a helpful tool for understanding the pathophysiology of brain development and its association with neurodevelopment in children born very preterm. Further studies are required to confirm a direct between FC and cognitive function.

## Methods

### Participants

The present study included children aged 6 years, born very preterm (< 32 weeks' gestation) and admitted to the NICU at Gachon University Hospital between 2010 and 2013**.** Cranial ultrasound scans were acquired serially during the hospital stay and at the term-equivalent age. Patients with major brain injuries (other than isolated grade I intraventricular hemorrhage on cranial ultrasound) or major disabilities, such as cerebral palsy, mental retardation, deafness, blindness, or congenital abnormalities, were excluded. All participants had normal hearing, vision, and motor development, as assessed at the serial follow-up. For preterm participants, perinatal data were derived from medical records. Healthy children born > 37 weeks' gestation were recruited from the local community for the control group. Based on a screening interview, the exclusion criteria for term-born children included major neurological impairment and MRI scanning incompatibility. All assessments were performed between December 2016 and April 2019. Depending on family preference, scans were performed either on the same day or as close as possible to their neurodevelopmental evaluation within one week. This study was approved by the institutional review board of Gachon University Gil Medical Center (GBIRB2016-239). All methods were carried out in accordance with the relevant guidelines and regulations. Informed consent was obtained from the parents of all participants.

### Neurodevelopmental assessment

Cognitive function at 6 years of age was assessed by an experienced psychologist who was blinded to the purpose of the study. The evaluation involved the use of the WISC-IV and the CBCL. The WISC-IV provides full-scale intelligence quotients, which indicate overall cognitive abilities, and four index scores based on specific cognitive profiles: verbal comprehension, perceptual reasoning, working memory, and processing speed. Each score is set to have a mean of 100 and standard deviation of 15 for the population as a whole. The CBCL assesses externalizing behavior problems, which are composed of attention problems and aggressive and delinquent behavior, and internalizing behavior, which comprises withdrawal, depressed behavior, and somatic complaints. The raw scores of the CBCL are reported as T-scores with a mean of 50 and SD of 10. Higher T-scores suggest more behavioral problems, and T-scores ≥ 60 are considered in the clinical range and indicate behavioral problem^52^.

### MRI data acquisition

DTI, rs-fMRI, and T1-magnetization-prepared rapid gradient-echo (MPRAGE) images of all the subjects were obtained using a 3.0-Tesla MR scanner (Verio, Siemens with a Siemens matrix coil) under the supervision of an attending pediatrician. The DTI was performed using the following parameters: b = 0 and 800 s/mm; repetition time (TR), 13,000 ms; echo time (TE), 76 ms; flip angle, 90°; pixel bandwidth, 1628 Hz/pixel; total acquisition time, 14 min 33 s; and iso-voxel resolution, 1.8 mm. The rs-fMRI imaging parameters used were as follows: TR = 3000 ms, TE = 30 ms, in-plane voxel resolution = 3.4 × 3.4 mm 2, field-of-view = 220 mm × 220 mm, total acquisition time = 5 min 36 s, and slice thickness = 3.4 mm. Data were collected continuously at 110-time points, and the scanning range was the whole- brain. The T1-MPRAGE imaging parameters used were as follows: TR, 1900 ms; TE, 2.93 ms; flip angle, 8°; pixel bandwidth, 170 Hz/pixel; matrix size, 256 × 208; field-of-view, 256 mm; NEX, 1; slice thickness, 1 mm; total acquisition time, 4 min 9 s. For successful scanning without sedatives, scans were scheduled around the child's natural nap time. If the child failed to sleep naturally, a low dose of chloral hydrate (30 mg/kg) was orally administered.

### DTI analysis

DTI images were processed using the FMRIB Software Library (FSL, Oxford, United Kingdom)^53^, including the eddy current correction and the Brunauer–Emmett‒Teller (Brain Extraction Tool) method^54,55^. Fractional anisotropy (FA) images of the DTI preprocessing results were used in the TBSS (part of the FSL)^56^. All FA images were aligned to a target in common space using our previously described methods^29,57^. Voxel-wise statistical analyses were performed using Randomise (part of the FSL)^58^. A correction for multiple comparisons and cluster formation was conducted using threshold-free cluster enhancement (TFCE). Voxels with *P* < 0.05 (TFCE-corrected) were considered significantly different.

### Functional network connectivity analysis

Analysis of all rs-fMRI data was performed using the CONN toolbox (www.nitrc.org/projects/conn), version 18b^16^. A detailed description of this toolbox has been provided elsewhere^59^. Before data processing, the first five-time points from the rs-fMRI data were excluded to allow magnetic equilibration. The preprocessing steps were included realignment to the first volume for head motion correction, outlier scrubbing, functional and structural segmentation, normalization to the pediatric template^60^, and smoothing by a kernel of 6 mm full width at half maximum. We then used the CONN toolbox to implement a denoising process of confounding factors using linear regression and band-pass filtering (0.008–0.09 Hz) to remove the subject's estimated motion parameters and other artificial effects, including BOLD signals in WM and cerebrospinal fluid regions, which were included as additional covariates. Between-group differences in FC were assessed at the network level. The ROI-to-ROI analysis was performed using CONN RSN nodes, which included 32 seeds/targets^61^. Results were reported at a height threshold false discovery rate of *P* < 0.05, with two-sided seed level correction and permutation tests.

### Statistical analyses

Demographic and neurodevelopmental data were compared between the preterm and term groups. Data are expressed as the number (%) or mean ± standard deviation. Univariate analyses of categorical variables were performed using the chi-square test, and the independent Student's *t-*test was used for continuous variables. Statistical significance was set at *P* < 0.05. To correct for multiple comparisons of the CBCL scores, the level of significance was set at *P* < 0.004, using the Bonferroni correction (11 comparisons). Statistical analyses were performed using the Statistical Package for the Social Sciences version 22.0 software (IBM Corporation, Armonk, NY, USA).

## Data Availability

The data that support the findings of this study are available from the corresponding author upon reasonable request.
